# Supplementary material for: The Significance of SPP1 in Lung Cancers and Its Impact as a Marker for Protumor Tumor-Associated Macrophages
Source: Cancers (Basel). 2023 Apr 12;15(8):2250. doi: 10.3390/cancers15082250 (PMC10136569; doi:10.3390/cancers15082250)
Supplement: Supplementary file 1 [file cancers-15-02250-s001.zip › cancers-2270634-supplementary.pdf]

Supporting Table S1; Published studies described on SPP1 expression in lung cancer.

Reference number	Histological type	pStage	SPP1 staining evaluation method	Number of cases	Comments on SPP1
[41]	Ad:65,SCC:122, Others:20	I:136, II-III:71	Positive area (<20%:low, ≧20%:high)	low:101, high:106	High SPP1 expression in Stage I NSCLC is associated with shorter OS and RFS.
[42]	Ad:115, Others:65	Unknown	Positive area (<60%:negative, ≧60%:positive)	negative:59, positive:121	SPP1 expression on primary lesion is not associated with bone metastasis.
[43]	Ad:55,SCC:102, Others:2	I:43, II-III:116	Positive area (0-3)＋Staining intensity(0-3) /2→>3: positive	negative:68, positive:91	The combination of SPP1 and CD44v6 is associated with shorter OS and RFS.
[44]	Ad:128,SCC:58, Others:24	I:135, II-III:75	0:no staining, 1:intermediate, 2: strong	0:44, 1:125, 2:22	Low SPP1 was associated with shorter RFS.
[45]	Ad:49, SCC:43	III-IV	Positive area(0-100%), Staining intensity(0-3)	negative:58, positive:34	SPP1 expression is an independent predictor of response to platinum-based chemotherapy and of the prognosis.
[46]	Ad:71, SCC:92	I:44, II-IV:119	Positive area (0-3)＋Staining intensity(0-3) /2→>3: positive	negative:54, positive:109	SPP1 positive group in Stage I-II NSCLC was associated with shorter OS and RFS.
[47]	Ad:34, SCC:39 (pStageIII-IV)	I-II:28, III-IV:73	Positive area (<30%:negative,≧30%:positive)	negative:59, positive:42	SPP1 may be able to predict poor prognosis and cisplatin resistance in patients.
